# Identification of 3′,4′-Dimethoxy Flavonol-3-β-d-Glucopyranoside Metabolites in Rats by Liquid Chromatography-Electrospray Ionization Ion Trap Mass Spectrometry

**DOI:** 10.3390/molecules21040470

**Published:** 2016-04-09

**Authors:** Yuan Zhu, Jun Wen, Yuqing Cao, Yuanying Jiang, Jinghua Huang, Guorong Fan, Yuefen Lou

**Affiliations:** 1 Shanghai Institute of Technology, Shanghai 201418, China; zy61420@outlook.com; 2 Shanghai Key Laboratory for Pharmaceutical Metabolite Research, School of Pharmacy, Second Military Medical University, Shanghai 200433, China; wenjunapple@163.com (J.W.); caoyuqing1109@163.com (Y.C.); 3 Center for New Drug Research, School of Pharmacy, Second Military Medical University, Shanghai 200433, China; 13761571578@163.com; 4 Shanghai 8 Plus 1 Pharmaceutical Technology Company Limited, Shanghai 200233, China; huangjh.81@188.com; 5 Department of Clinical Pharmacy, Shanghai General Hospital, School of medicine, Shanghai Jiao Tong University, No. 100 Haining Road, Shanghai 200080, China; 6 Department of Pharmacy, Branch of Shanghai First People’s Hospital, Shanghai 200081, China

**Keywords:** LC-ESI/MS^n^, 3′,4′-dimethoxy flavonol-3-β-d-glucopyranoside, metabolites, metabolic pathway

## Abstract

A method using liquid chromatography-electrospray ionization ion trap mass spectrometry was established for the identification of metabolites in feces, urine and bile in rats after oral administration of 3′,4′-dimethoxy flavonol-3-β-d-glucopyranoside (abbreviated DF3G). Seven metabolites in rat feces, urine and bile were firstly identified on the basis of their MS fragmentation behaviors. Three metabolites were identified in the feces, 6 in the urine and 2 in the bile, which suggested that demethylation, deglycosylation and deglycosylation followed by glucuronide conjugation were the major metabolic pathways for DF3G *in vivo*. Hydrolyzation might be the first step in the absorption and metabolism of DF3G. The possible metabolic pathway was proposed for the first time. The established method was simple, reliable and sensitive, revealing that it could be used to rapidly screen and identify the structures of metabolites of DF3G to better understand its metabolism *in vivo*.

## 1. Introduction

**Figure 1:**
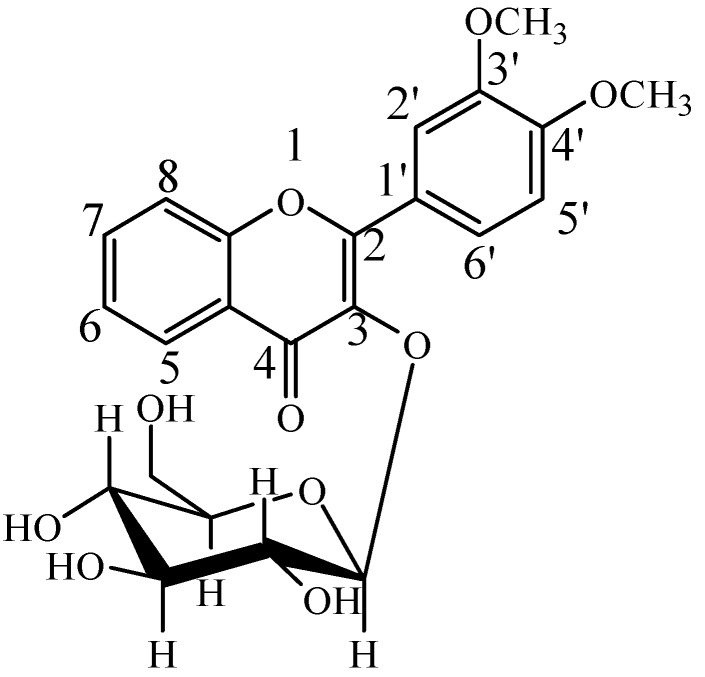
Chemical structure of DF3G.

Flavonoids act as various functional secondary metabolites in plants [1]. More than 4000 flavonoids, such as flavonols, flavones, flavanols, flavanonols, flavanones, and isoflavones have been reported in edible plants and are consumed regularly in the human diet [2]. Flavonoid compounds share the same basic skeleton, the flavan-nucleus, consisting of two aromatic rings with six carbon atoms (ring A and B) interconnected by a hetero cycle including three carbon atoms (ring C) [3,4,5,6]. Flavonoids have appealing pharmacological activities, such as antioxidant, anti-allergic, antibacterial, anti-inflammatory, antimutagenic and anticancer effects [7]. Recent studies suggested that flavonoids prevent experimental hepatic steatosis, dyslipidemia and insulin sensitivity primarily through inhibition of hepatic fatty acid synthesis and increased fatty acid oxidation [8]. For example, quercetin, a flavonol compound, could lower the risk of cardiovascular disease via strongly up-regulated low density lipoprotein receptor gene expression in hepatic cells [8]. DF3G (Figure 1), 3′,4′-dimethoxy flavonol-3-β-d-glucopyranoside, is synthesized on the basis of flavonol and there has been an application for a Chinese patent [9]. Pharmacodynamics research has demonstrated that DF3G could significantly reduce not only blood lipids but also aortic atherosclerotic lesions and liver injury induced by carbon tetrachloride in mice [9].

The study of drug metabolism in the preclinical phase of drug discovery is a critical issue. The metabolic clearance *in vivo* has a relationship with drug bioavailability and half-life. Metabolite identification could provide important information to help understand the comprehensive metabolic pathways of drug sin rats, and would be useful for subsequent studies on their metabolism in other species. Our previous work illustrated the low cumulative excretion rate of DF3G in rats after oral administration, so this study focused on *in vivo* metabolites of DF3G in rat feces, urine and bile.

Liquid chromatography-mass spectrometry (LC-MS) has become the most popular analytical technology for the qualitative and quantitative analysis of metabolites in biological matrices with high sensitivity and low consumption of samples [10,11,12]. The ion trap mass spectrometry is composed of an ion source and quadrupole ion trap mass analyzer. The reproducibility of the product ion spectra make this technique extremely effective in the recognition of unknown compounds [13]. Though high resolution mass spectrometry has a huge advantage in metabolite identification, ion trap mass spectrometry could identify metabolites on the basis of the fragmentation pattern of the original drug as well, and the poor sensitivity will be offset by pretreatment.

Many studies have reported the metabolic pathway of flavonoids [14,15,16]. The present study aims to elucidate the metabolic pathways of DF3G, a new synthetic flavonoid, after oral administration, which provided a practical strategy for rapidly screening and identifying metabolites of DF3G in a biological sample by the use of liquid chromatography-electrospray ionization ion trap mass spectrometry (LC-ESI/MS^n^). The parent drug and its seven metabolites were firstly found in rat feces, urine and bile, which will be useful for future studies.

## 2. Results and Discussion

### 2.1. Optimization of Chromatography and MS Conditions

In order to achieve an optimal peak shape and good separation, the stationary phase, the composition of mobile phase and elution programs were evaluated. Based on their polarity and acidity properties, various available columns of different bonded phases were carefully investigated. As a result, the Diamonsil C18(2) (150 mm × 4.6 mm, 5 μm) column was chosen for its high column efficiency and peak symmetry. Different mobile phase were attempted and the results showed that the addition of formic acid not only could remarkably better the shape of peak, but also improve the efficiency of ionization, which was beneficial to MS analysis. Meanwhile, acetonitrile was used as the organic phase, with better peak symmetry than that of methanol. Therefore, the mobile phase was selected as 0.2% formic acid-water (A) and 0.2% formic acid-acetonitrile (B). The final chromatography parameters are shown in Section 3.6.

ESI in both positive and negative ion modes were tried and the results showed that ESI in positive mode was more sensitive for DF3G and its metabolites in the present study. The instrumental parameters were optimized by analyzing the metabolites for the maximum intensity. The optimized parameters in the positive ion mode were as follows: ion spray voltage, 4000 V; sheath gas, 35 arbitrary units; auxiliary gas, 5 arbitrary unit; capliary temperature, 350 °C; capillary voltage, −25 V; tube lens offset, 80 V. To obtain the most abundant information for all the constituents in rat biosamples, the data-dependent scan was used in this analysis. The two most abundant ions in each scan were selected and subjected to MS^n^ analysis and the relative collision energy for CID was set at 35%, which could produce satisfactory MS^n^ fragmentation information [17].

### 2.2. Identification of DF3G

A Retro-Diels-Alder (RDA) reaction is the main fragmentation process on the C ring of flavone aglycones [18]. Various conjugated flavone aglycones systems influenced the fragmentation behavior of C ring. The substitution patterns on A and B lead to different types of product ions and the intensity of relative abundances, respectively [18,19]. These findings will be helpful in identifying the structures of flavones rapidly with the method of mass spectrometry.

**Figure 2:**
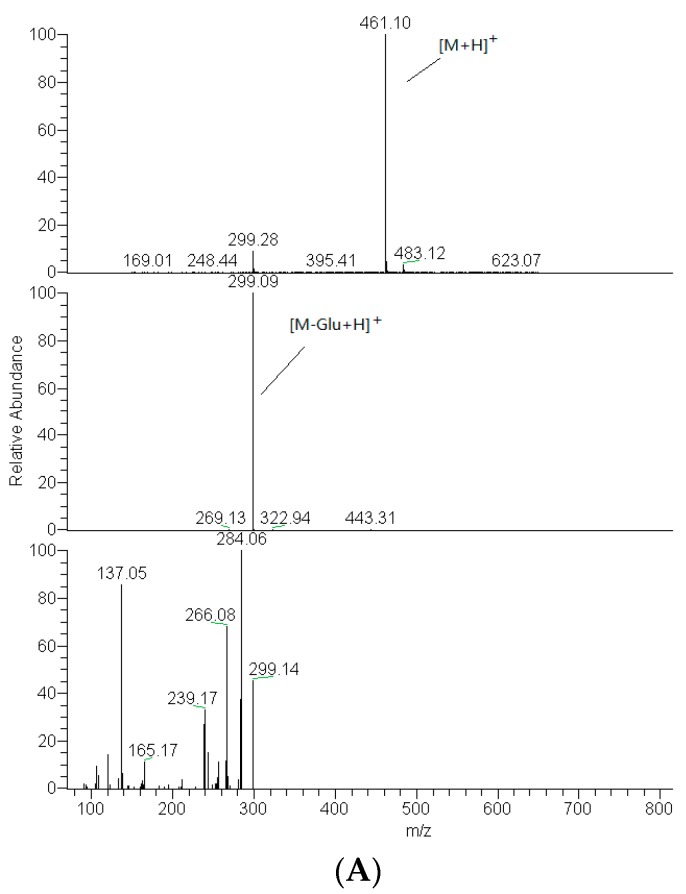

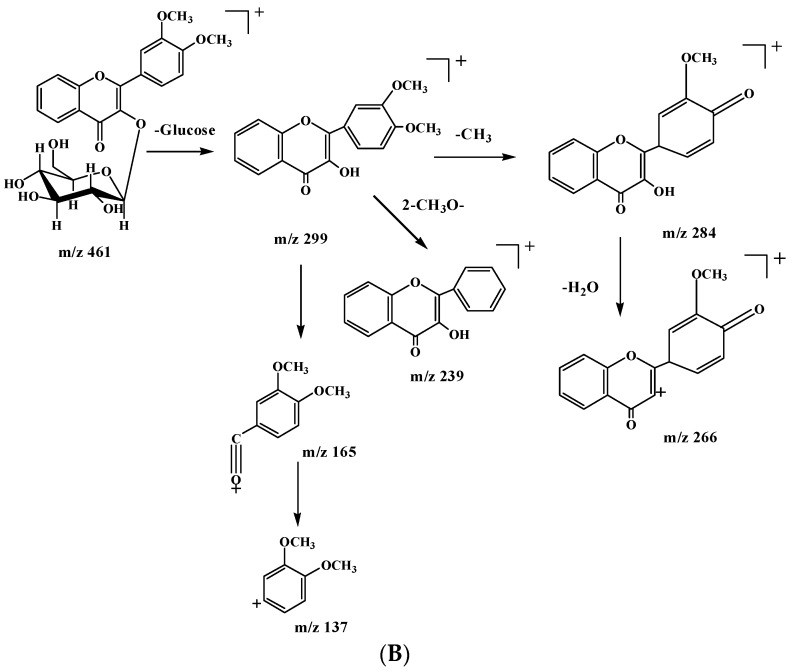
Mass spectrum (**A**) and fragmentation pathway (**B**) of DF3G in positive ion mode.

The [M + H]^+^ ions of DF3G in positive mode were *m*/*z* 461. The MS^2^ spectrum showed an ion at *m*/*z* 299, 162Da less than parent drug, corresponding to the loss of dehydrated glucose [20]. And the MS^3^ spectrum showed ions at *m*/*z* 284, 266, 239, 165 and 137. The *m*/*z* 284 and *m*/*z* 239 were fragmented by loss of -CH_3_ and two -CH_3_O- on B ring, then the *m*/*z* 266 was formed on the basis of *m*/*z* 285 by loss of H_2_O. Two fragment ions, *m*/*z* 165 and *m*/*z* 137, generated because of RDA reaction on the C ring of *m*/*z* 299. The mass spectrum and fragmentation pathway of DF3G in positive ion mode are shown in Figure 2 [21].

The mass fragmentation pathway of DF3G in positive ion mode could offer a basis to deduce the metabolites. In this study we were able to determine that the RDA reaction happening on the C ring of DF3G had to meet the demand of losing glucoside, which suggested that DF3G offered better protection to the maternal structure of flavone because of the glucoside.

### 2.3. Identification of Metabolites

The metabolic transformation of drugs *in vivo* occurs mainly by way of the reaction of functional groups themselves or a binding reaction with endogenous molecules. This means that the metabolite still retains the basic framework structure or substructure of the original drug. Therefore, metabolites undergo fragmentation in mass spectrometry or neutral loss with the characteristics of the original drug.

**Figure 3:**
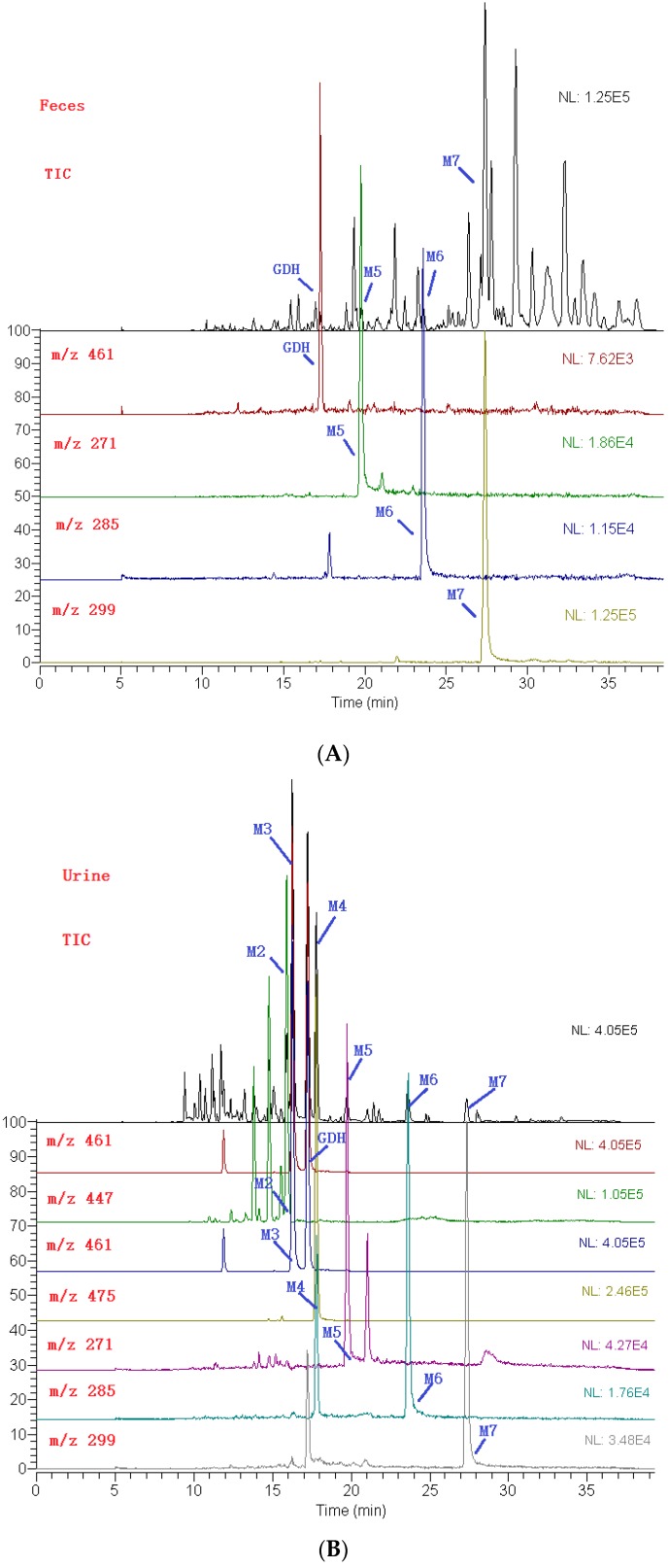

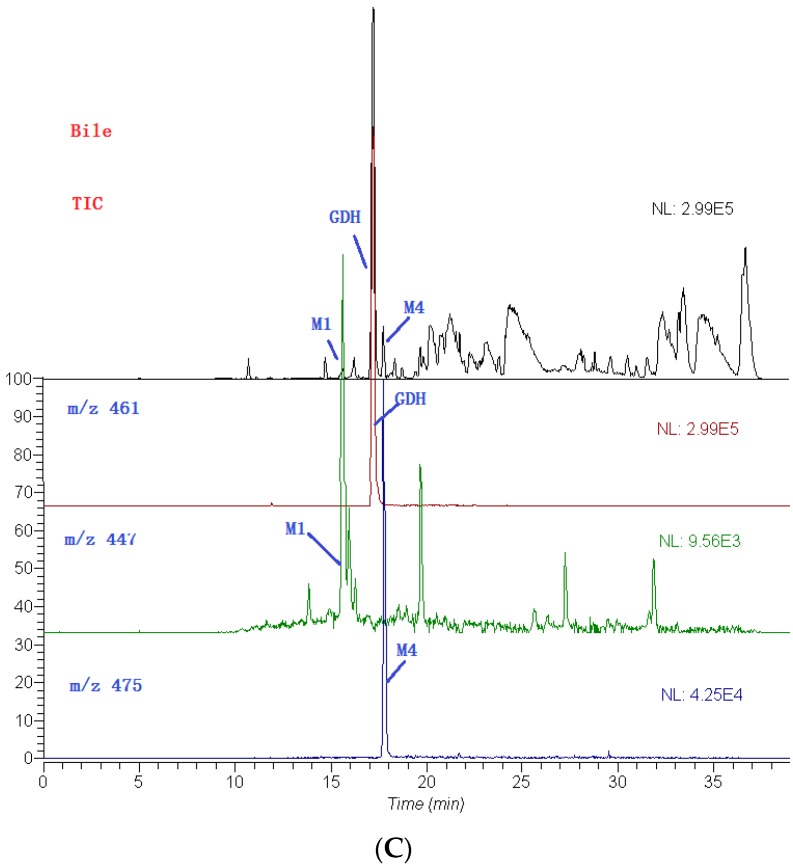
Extracted ion chromatograms for rat metabolites of DF3G in feces (**A**); urine (**B**) and bile (**C**) by LC-ESI/MS^n^ analysis.

**Figure 4:**
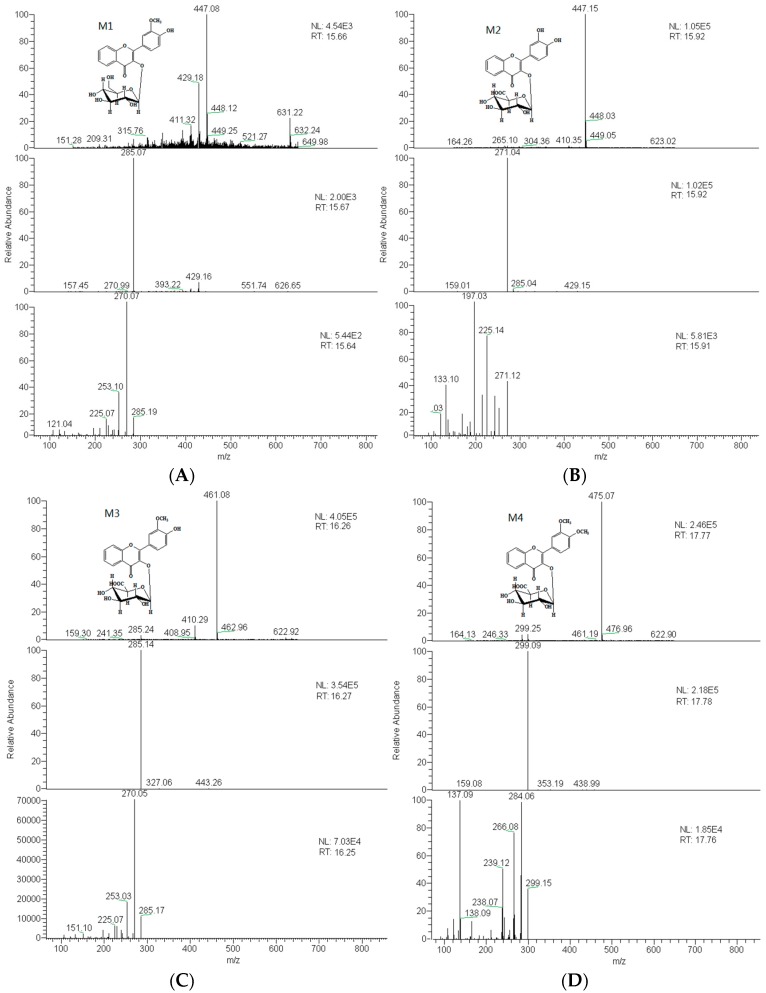

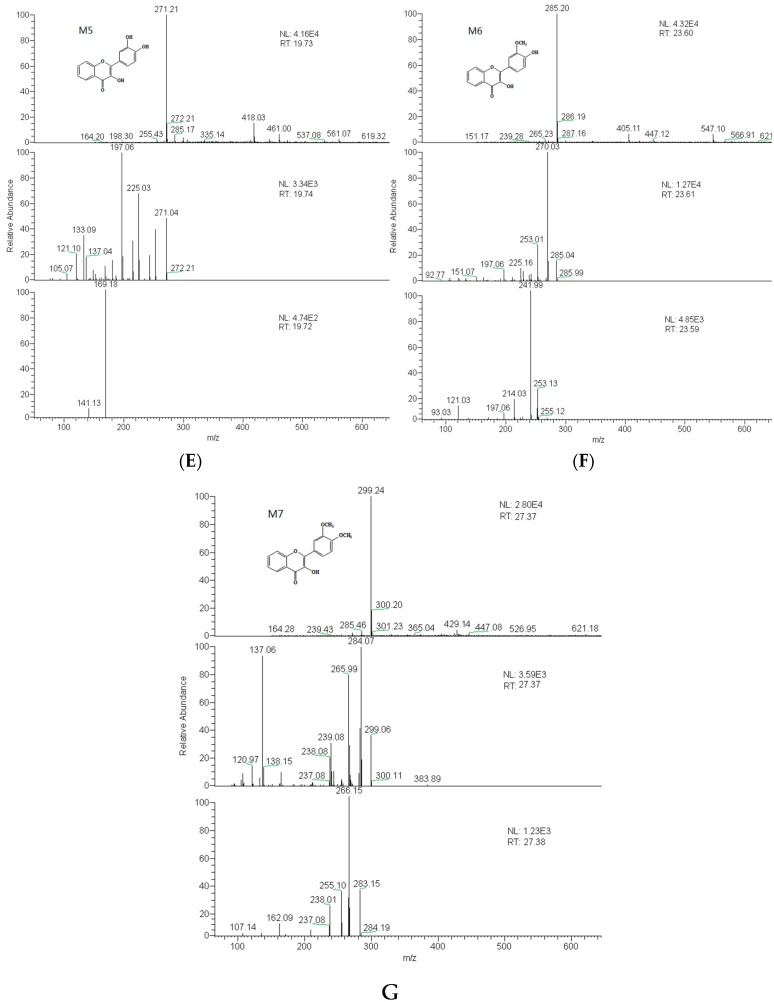
The mass spectra of metabolites in positive ion mode of M1 (**A**); M2 (**B**); M3 (**C**); M4 (**D**); M5(**E**); M6 (**F**) and M7 (**G**).

By extracting ion information and comparing with blank biological sample under the condition of “3.6”, “3.7”, the prototype components and seven metabolites were detected and inferred. Based on the structure characteristics of DF3G, proposed metabolites are given in Table 1. Figure 3 shows the extracted ion chromatograms for rat metabolites of DF3G by LC-ESI/MS^n^ analysis. Figure 4 shows the mass spectra of metabolites in positive ion mode.

The mass spectra of M1, which was observed in bile at a retention time of 15.6 min, showing higher polarity compared with DF3G, showed an [M + H]^+^ at *m*/*z* 447, which was 14 Da less than the protonated molecular ion of DF3G. The protonated product ion series of M1 were *m*/*z* 285, 270. The *m*/*z* 285 was fragmented by loss of 162 (dehydrated glucose) and *m*/*z* 270 was from the loss of 15 (CH_3_) afterwards, which was in agreement with the DF3G. 4′-methoxyl was more easily catalyzed than 3′-methoxyl [22]. It was deduced to be a demethylated metabolite of DF3G. Thus, M1 was identified as 3′-methoxy-4′-hydroxy flavonol-3-*O*-β-d-glucopyranoside (Figure 4A).

Metabolite M2 observed in the rat urine formed a protonated molecular ion [M + H]^+^ at *m*/*z* 447, which was 14 Da less than DF3G. The retention time was 15.9 min. The fragment ions of M2 were *m*/*z* 271, 225, 197. The precursor ion of M2 was the same with that of M1; however, neither the retention time nor the fragment ions of them were identical. Thus M1 and M2 might be thought to be different metabolites. The major fragment ion of *m*/*z* 271 was 176 Da less than protonated molecular ion of M2 and was produced by the loss of C_6_H_8_O_6_, indicating that glucuronide conjugation happened on the C ring [20,23]. This information revealed that M2 was formed by the pathway of deglycosylation, demethylation and glucuronide conjugation. M2 was also deduced to be 3′,4′-dihydroxy flavonol-3-*O*-β-d-glucuronide (Figure 4B).

Metabolite M3 was only observed in urine with a retention time of 16.2 min. M3 exhibited an [M + H]^+^ ion at *m*/*z* 461, the same precursor ion with the parent drug. The major fragment ions *m*/*z* 285 ([M − C_6_H_8_O_6_]^+^), 270 ([M − C_6_H_8_O_6_ − CH_3_]^+^) were identical to those of M1, suggesting that the structures of M1 and M3 were similar [22]. These data revealed that M3 was a glucuronide-conjugated product after the deglycosylation and demethylation of DF3G. M3 was inferred as 3′-methoxy-4′-hydroxy flavonol-3-*O*-β-d-glucopyranoside (Figure 4C).

Metabolite M4 was detected in feces and urine with retention time of 17.8 min and produced an [M + H]^+^ ion at *m*/*z* 475, which was 14 Da higher than that of DF3G. Upon collision-induced dissociation, the [M + H]^+^ ion at *m*/*z* 475 produced fragment ions at *m*/*z* 299 ([M − C_6_H_8_O_6_]^+^), 284 ([M − C_6_H_8_O_6_ − CH_3_]^+^) and 266 ([M − C_6_H_8_O_6_ − CH_3_ − H_2_O]^+^). The fragmentation pathways of M4 and DF3G were similar, and its molecular mass was 14 Da higher than DF3G because of the change of glycosyl. Based on the above analysis, M4 was deduced to be 3′,4′-dimethoxy flavonol-3-*O*-β-d-glucopyranoside (Figure 4D).

Metabolite M5 observed at a retention time of 19.7 min was detected in feces and urine, showing lower polarity compared with DF3G. It produced the [M + H]^+^ ion at *m*/*z* 271, which was 176 Da less than the precursor ion of M2 and 190 Da less than the precursor ion of DF3G. The protonated product ion series of M5 were *m*/*z* 225, 197, 169, the same with those of M2, which indicated that they had similar structure. These data showed M5 might be formed by concerted deglycosylation (C_6_H_10_O_5_) and demethylation (2CH_3_) of the parent drug and then produce a glucuronide-conjugated metabolite, M2 [20]. M5 was identified as 3′,4′-dihydroxy flavonol (Figure 4E).

Metabolite M6 was observed in feces and urine with retention time of 23.6 min, producing the protonated molecular ion at *m*/*z* 285, 176 Da less than that of DF3G. The [M + H]^+^ ionat *m*/*z* 285 produced fragment ions at *m*/*z* 270, 242. The similar fragment ions with M1 and M3 concealed its structure. Thus M6 was tentatively identified as deglycosylation (C_6_H_10_O_5_) and demethylation (CH_3_) product of DF3G [20,22]. M6 was deduced to be 3′-methoxy-4′-hydroxy flavonol (Figure 4F).

Metabolite M7 was detected in feces and urine at a retention time of 27.4 min, and gave the [M + H]^+^ ion at *m/z* 299, 162 Da less than the precursor ion of DF3G. The protonated product ion series of M7 were *m/z* 284 ([M − CH_3_]^+^) and 266 ([M − CH_3_ − H_2_O]^+^). The fragmentation pathway was similar to that of the parent drug and M4. Based on previous analysis, M7 was identified as deglycosylation (C_6_H_10_O_5_) metabolite of DF3Gand it was deduced as 3′,4′-dimethoxy flavonol (Figure 4G) [20]. When DF3G loses a molecule of glucose, the lower polarity might result in the longer retention time of M7 than that of DF3G in the revised-phase chromatographic system.

### 2.4. A Summary of Metabolic Pathways for DF3G

**Figure 5:**
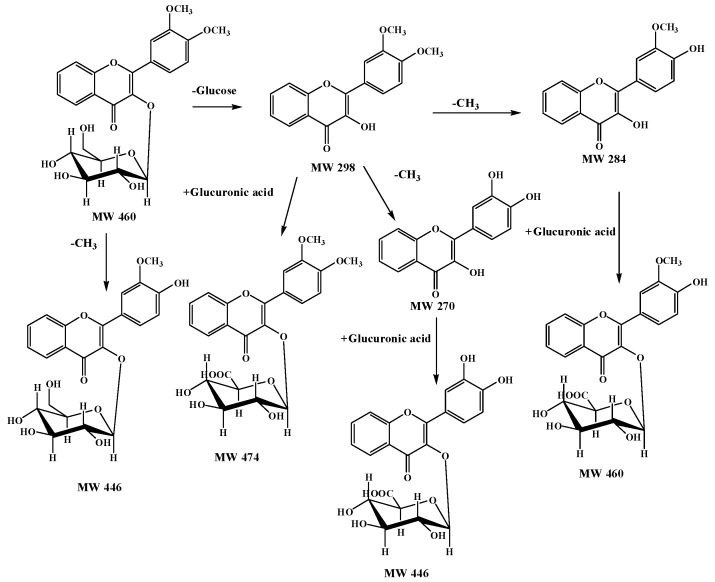
Possible metabolic pathways of DF3G in rats.

Based on the above analysis, DF3G undergoes demethylation (M1), deglycosylation and didemethylation followed by glucuronide-conjugation (M2), deglycosylation and demethylation followed by glucuronide-conjugation (M3), deglycosylation followed by glucuronide-conjugation (M4), deglycosylation and didemethylation (M5), deglycosylation and demethylation (M6) and deglycosylation (M7) reactions in rats. Among these, a total of 3 metabolites were identified in the feces, 6 were detected in the urine and 2 were found in the bile, which suggested that demethylation, demethylation + deglycosylation and deglycosylation followed by glucuronide conjugation were the major *in vivo* metabolic pathways for DF3G. The possible metabolic pathways of DF3G in rats are proposed, as shown in Figure 5.

## 3. Experimental Section

### 3.1. Chemicals and Reagents

The standard, DF3G, was offered by Department of Organic Chemistry of Second Military Medical University. The purity of the constituents was no less than 99.0% according to HPLC analysis. Acetonitrile and formic acid were HPLC grade and purchased from TEDIA Company (Fairfield, CA, USA). Methanol was HPLC grade and purchased from Merck Company (Darmstadt, Germany). The water for the experiment was deionized water made by Hi-Tech (Shanghai, China) water purification system (18.2 M).

### 3.2. Preparation of Standard Solutions

The appropriate amounts of the standard (DF3G) was prepared by accurately weighing the required amounts into volumetric flasks and dissolved in methanol. The stock solutions is stored at −20 °C before analysis.

### 3.3. Animals and Drug Administration

Twelve healthy SD rats, weighing 200 ± 10 g, half male and half female, were supplied by Shanghai Slac Laboratory Animal and fed with certified standard diet and tap water *ad libitum*. Temperature and humidity were regulated at 21–24 °C and 30%–60%, respectively. A 12 h cycle of light and darkness was established. After 1 week of acclimatization, they were randomly divided into 2 groups (3 males and 3 females for each group). Rats were fasted for 12 h before receiving oral administration of DF3G (dissolved in 0.5% CMC-Na) at a dose of 50 mg/kg body weight. Animal had free access to water and food 4 h after drug administration. Rats in group one were housed in metabolic cages for the collection of feces and urine samples after oral administration of DF3G. And the feces and urine samples were collected for 72 h and 48 h, respectively. For the collection of bile samples, rats were under anesthetized with ethyl carbamate after being administered. The common bile duct was cannulated with polypropylene tubes for the collection. The samples were collected for 24 h. And all the samples were frozen in polypropylene tubes at −60 °C until analysis.

### 3.4. Preparation of Urine, Feces and Bile Samples

The feces samples were dried in the air and then ground, weighed and homogenated with 1 mL of 80% acetonitrile–water. After centrifuging at 4000 rpm for 10 min, the supernatant of feces homogenate was transferred to a clean tube and evaporated to dryness at 35 °C under vacuum with SpeedVac Concentrator (SPD131DDA, ThermoFisher, Waltham, MA, USA). The residues were reconstituted with 200 microliter of methanol–water (80:20, *v*/*v*) and the mixture was vortexed for 3 min and then centrifuged at 12,000 rpm for 10 min. Finally, a 10 microliter aliquot of the supernatant was injected into the LC-ESI/MS^n^ system for analysis.

1 mL urine samples applied to a conditioned SPE column (LC-C18, 500 mg, SUPELCO, Bellefonte, PA, USA), which was washed with 4 mL of water and 4 mL of 5% methanol. The metabolites were successively eluted with 2 mL of methanol. The eluate was evaporated to dryness at 35 °C. The residues were reconstituted with 200 microliter of methanol–water (80:20, *v*/*v*) and prepared for the injection, the same as the feces samples described above.

1.5 mL acetonitrile was added to 0.5 mL bile samples. The mixture were vortexed for 3 min and centrifuged at 12,000 rpm for 10 min. 1.8 mL supernatant was transferred to a clean tube and evaporated to dryness at 35 °C. The residues were reconstituted with 200 microliter of methanol–water (80:20, *v*/*v*) and prepared for the injection, the same as the feces samples described above.

### 3.5. Instrumentation

The Dionex Ultimate 3000 liquid chromatography system (Thermo Fisher Company, Waltham, MA, USA) equipped with a quaternary solvent delivery system, an autosampler, a column compartment, and a DAD detector was used for all analyses, along with a Chromeleon 6.8 chromatographic working-station. The LCQ Fleet Mass Spectrometer (Thermo Fisher Company, Waltham, MA, USA) equipped with an ESI ion source, and an ion trap mass analyzer with Xcalibur™ 2.6.0 Data Working Station for data acquisition and processing.

### 3.6. Liquid Chromatographic Conditions

The chromatographic separation was carried on a Diamonsil C18(2) (150 mm × 4.6 mm, 5 μm) column at a column temperature of 25 °C. The auto-sampler temperature was kept at an ambient temperature of 25 °C. The mobile phase consisted of 0.2% formic acid-water (A) and 0.2% formic acid-acetonitrile (B), with a linear-gradient elution at a flow rate of 1 mL/min. The elution program was optimized and conducted as follows: 8% B (0–5 min); 8%–20% B (5–8 min); 20%–60% B (8–30 min); 60% B (30–35 min); 60%–8% B (35–35.1 min); 8% B (35.1–40 min). The postcolumn split ratio was 7:3, and the detection wavelength was 234 nm. The sample injection volume was 10 microliters.

### 3.7. Mass Spectrometer Conditions

For metabolite analysis, a full scan was run in the positive mode with a mass range from *m*/*z* 150 to 600 amu. The ESI at an ion spray voltage of 4000 V. The capillary temperature and voltage were maintained at 350 °C and 4V, respectively. The sheath gas and auxiliary gas were set at 35 arb and 5 arb, respectively. The tube lens was 80 V. The collision-induced dissociation energy was 35%.

## 4. Conclusions

In this experiment an LC-ESI/MS^n^ method was established to detect the metabolites in feces, urine and bile samples of rats orally administrated DF3G, a new synthetic flavonoid. DF3G could be detected in all biological samples, and a total of seven metabolites and their structures were elucidated based on the retention times in the revised-phase HPLC system and characteristic fragment ions. The main metabolic pathways were deglycosylation by breaking glucosidic bonds, demethylation and glucuronidation. DF3G was most likely hydrolyzed by two intestinal β-glucosidases, lactase phlorizin hydrolase (LPH) and cytosolic β-glucosidase enzyme (CBG), after oral administration and converted to its aglycone (M7) [24,25,26,27]. Hydrolyzation was the first step in the absorption and metabolism of dietary flavonoid glycosides. Then it might be catalyzed by microsomal enzymes and form demethylated metabolites (M5,M6), followed by a reconjugation step in the intestinal cell and/or in the liver with glucuronic acid after absorption of aglycone, generating their glucuronide-conjugated products (M2,M3,M4) [27]. DF3G, which was absorbed in blood directly without metabolism in intestines, was likely to produce demethylated metabolite (M1). Investigation into the metabolism of DF3G *in vitro* would be performed to confirm the proposed metabolic pathways *in vivo* including the prediction of the metabolism of DF3G in other species.

## Figures and Tables

**Table 1:** Mass spectrometry (MS^n^) data of metabolites of DF3G.

No.	t_R_ (min)	Compound Name	MW	Formula	Parent Ions (*m*/*z*)	MS^n^ (*m*/*z*)
M1	15.6	3′-methoxy-4′-hydroxy flavonol-3-*O*-β-d-glucopyranoside	446	C_22_H_22_O_10_	447	285, 270
M2	15.9	3′,4′-dihydroxyflavonol-3-*O*-β-d-glucuronide	446	C_22_H_22_O_10_	447	271, 225, 197
M3	16.2	3′-methoxy-4′-hydroxy flavonol-3-*O*-β-d-glucopyranoside	460	C_23_H_24_O_10_	461	285, 270
M4	17.8	3′,4′-dimethoxy flavonol-3-*O*-β-d-glucopyranoside	474	C_23_H_22_O_11_	475	299, 284, 266
M5	19.7	3′,4′-dihydroxy flavonol	270	C_15_H_10_O_5_	271	225, 197, 169, 270, 242
M6	23.6	3′-methoxy-4′-hydroxy flavonol	284	C_16_H_12_O_5_	285	270, 242
M7	27.4	3′,4′-dimethoxy flavonol	298	C_17_H_14_O_5_	299	284, 266
